# Does Excision of Heterotopic Ossification of the Elbow Result in Satisfactory Patient-Rated Outcomes?

**DOI:** 10.5704/MOJ.1703.017

**Published:** 2017-03

**Authors:** KN Sandeep, G Suresh, B Gopisankar, N Abhishek, A Sujiv

**Affiliations:** Department of Orthopaedics, Jawaharlal Institute of Postgraduate Medical Education and Research, Pondicherry, India

**Keywords:** heterotopic ossification, elbow joint, ankylosis, ulnar nerve, pronation, supination

## Abstract

**Introduction:**

Treatment of heterotopic ossification (HO) of the elbow is challenging and fraught with complications. Patients who sustain direct trauma to the elbow joint, the central nervous system, and thermal burns are at increased risk for development of HO. There is a paucity of studies and reports on patient’s self-evaluation after the excision of the heterotopic ossification.

**Materials and Methods:**

This retrospective study assessed outcomes after excision of heterotopic ossification around the elbow in a cohort of ten patients operated from 2012 to 2015. The outcome assessment was done by the Mayo Elbow Performance index (MEPI) and the American Shoulder and Elbow Surgeons-Elbow score (ASES-E scores).

**Results:**

The mean follow-up was 18.11 months after the operation. The Mayo Elbow Performance Score was excellent in two elbows, good in six and fair in two. The mean gain in flexion-extension arc after excision of HO was 80 degrees. All of the patients had residual flexion deformity postoperatively. Eight of the nine patients were able to do activities requiring flexion at final follow-up.

**Conclusion:**

Excision of HO around the elbow is associated with satisfactory patient-rated outcomes in spite of failure to regain full range of motion.

## Introduction

Heterotopic ossification (HO) of the elbow joint results from the formation of mature lamellar bone in extra-osseous tissues. The resulting limitation of elbow movements can range from mild to complete ankylosis. Patients who sustain direct trauma to the elbow joint, the central nervous system, and thermal burns are at an increased risk for development of HO^1^.

The incidence of HO following elbow trauma ranges from 0% to 49% following distal humerus fracture and 4% to 18% following ulnar-humeral dislocations^2^. The published clinical studies are centered on the measurement of elbow range of motion after the operation. There is a paucity of studies and reports on the patient’s self-evaluation after the excision of the HO. Hence, we did a study on patient-rated outcomes and elbow range of motion following excision of HO of the elbow joint.

## Materials and Methods

The hospital records were searched for patients who underwent excision of the HO around the elbow joint from January 2012 to May 2015. We identified nine patients (ten elbows) operated for HO during the study period. Patients identified were requested to come to the outpatient department for assessment of their outcomes. All patients reported for assessment; no patient lost to follow-up. The HO was classified as per the scheme proposed by Hastings *et al*^3^.

**Fig. 1:**
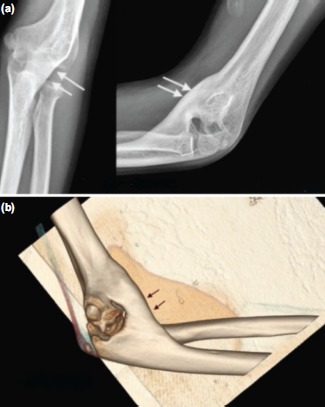
(a) Antero-posterior and Lateral radiographs showing HO Hastings IIIA bridging the humero-ulnar joint anteromedially (arrows), (b) 3D CT of case shown in Figure 1a.

**Fig. 2:**
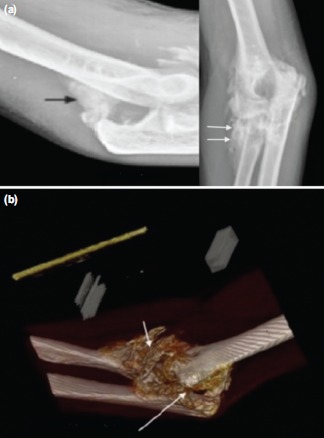
(a) Antero-posterior and lateral radiographs showing HO Hastings IIIC bridging the humero-ulnar (arrows) and radio-ulnar joints (arrows), (b) 3D CT of case shown in Figure 2.

Preoperative assessment included an anteroposterior and lateral radiographs of the affected elbow joint (Fig. 1a and 2a) and a computed tomography scan (CT) with three dimensional (3D) reconstruction (Fig. 1b and 2b).

**Fig. 3:**
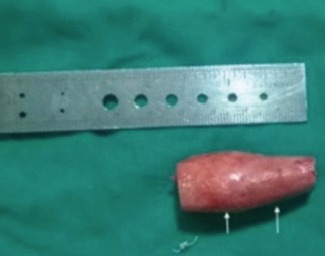
Photograph of excised bone of HO at the time of operation.

Surgery for the excision of the HO was performed under general anesthesia. Cases were operated under the tourniquet control for a clear operating field. The surgical approach was individualized for every patient depending on the anatomic location and extent of the HO. Either the medial approach or the lateral approach or a combination of two was used depending on the location of the HO in all except one patient. One patient required the midline anterior approach with the protection of the neurovascular bundle. The ulnar nerve was routinely transposed anteriorly. Radial nerve was found encased in one of the cases at the distal humerus and decompressed during surgery. An initial capsulotomy was done in all the cases to excise the ectopic bone (Fig. 3). The collateral ligaments were identified and protected during the operative procedure. Medial collateral ligament required repair in one of the case. The elbow was stabilized with a trans-olecranon pin until the healing of the medial collateral ligament. Radial head required excision in that case. At the conclusion of the procedure, haemostasis was achieved by diathermy. Bleeding cancellous bone surface was treated with the bone wax. The elbow joint was irrigated, and closure was done in layers over a suction drain to preclude hematoma collection in the post-operative period.

**Fig. 4:**
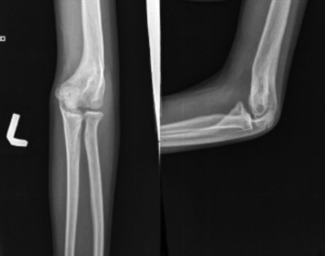
Post-operative antero-posterior and lateral radiograph of the case above in Figure 1 after 12 months of operation.

**Fig. 5:**
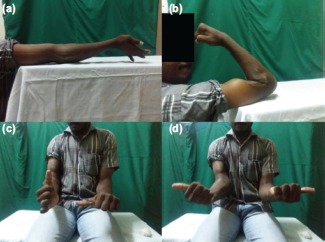
Elbow range of motion of the case shown in Figure 2 after 12 months of operation (a) Extension, (b) Flexion, (c) Pronation and (d) Supination.

After the operation, Oral Indomethacin 25 mg three times daily was started for six weeks for prophylaxis against HO recurrence. The dose was adjusted for patients less than 18 years. The suction drain was discontinued after 48 hours of operation. Pain control was obtained by opiates with dose adjusted as per weight. Patients were put on continuous passive motion machine after 24 hours of operation for eight hours per day and continued for one week. Active assisted, and passive movements of the elbow were started. Active physiotherapy was started one week after operation and patients were followed up at regular intervals. Radiographs of the patients were taken at regular intervals (Fig. 4). One patient needed a deviation from protocol because of injury to the medial collateral ligament. The Elbow was stabilized by a trans-olecranon pin in the case above, and mobilization of the elbow was started after three weeks. Follow-up range of motion of the case above is shown in Fig. 5.

The outcome assessment was done by the Mayo elbow performance index (MEPI) and the American Shoulder and Elbow Surgeons-Elbow score (ASES-E scores)^4^.

## Results

Nine patients with ten elbows reported for assessment. One patient had both elbows with HO. The mean follow-up was 18 months (12 - 26 months). The demographic data is summarized in Table I. The functional and objective outcome data are summarized in Table II. The Mayo elbow performance score (Table II) was excellent in two, good in six and fair in two elbows. The mean MEPI was 81. The gain of flexion/extension arc was at a mean of 80 degrees. The gain of pronation and supination from mid-prone position was at a mean of 51.5 and 73 degrees respectively degrees. The mean value for patient’s satisfaction after surgery was 6.7 on a scale of ten (Table II). The median Grip strength measured by dynamometer was decreased in five patients by a mean of 30 percent. Grip strength was equal in three patients. The grip strength couldn’t be compared in one patient because of elbow HO on both the sides. All elbows had residual flexion deformity in the post-operative period. There was a residual flexion deformity of 10 degrees in two, 20 degrees in two, 30 degrees in five and 40 degrees in one elbow after the operation. The mean values for intensity of pain on lifting heavy weight (5KG) and pain on doing repeated elbow movements were 4.62 and 3.75 of 10 respectively. One patient failed to gain any movement after operation due to recurrence of HO. One patient sustained radial nerve palsy which recovered. He also sustained deep infection which healed on debridement and antibiotic therapy. Eight of the nine patients could do activities requiring flexion (comb hair and do up button of shirt). All patients could reach the perineum. Nine of nine patients could manage to tie the shoe. No patient had elbow instability at follow-up.

**Table I:** Demographic data, cause of HO and duration of follow-up

Patient number	Elbow Hastings class	^a^ Age (years)	^b^ Sex	Side	Time to operation after HO (months)	Cause of HO	Follow-up (months)
1	^c^ IIIA ^c^ IIA	50	M	Right Left	2	^d^ Long recumbency (on life support after snake bite)	16
2	IIIA	24	M	Left	26	Non-specific elbow trauma	12
3	IIA	30	M	Right	4	Elbow dislocation	12
4	IIA	15	M	Left	5	Intercondylar fracture of distal humerus	18
5	IIIA	23	F	Right	12	Elbow trauma	20
6	IIC	29	M	Right	16	Elbow trauma	22
7	IIIA	5	F	Left	24	Supracondylar fracture distal humerus	13
8	IIA	43	M	Left	14	Elbow dislocation	24
9	IIIC	43	M	Right	24	Elbow dislocation	26

a Mean age of patients was 38.7 years.

b 7 Males and 2 Females.

c Patient 1 had both elbows with HO.

d Patient 1 was on ventilator support for 56 days after a snake bite.

**Table II:** Elbow range of motion and patient rated satisfaction

Patient number	Elbow Hastings class	^a^ Patient rated satisfaction	^b^ MEPI	^c^ Flexion/Extension in degrees Min	^d^ Flexion/Extension in degrees Max	^e^ Pronation	^f^ Supination
1	IIIA	10	80	20	110	70	85
	IIA	8	80	30	120	65	80
2	IIIA	3	70	30	30	0	0
3	IIA	4	80	10	110	0	80
4	IIA	5	80	30	120	70	85
5	IIIA	8	85	30	130	65	90
6	IIC	6	90	20	110	55	70
7	IIIA	6	65	40	110	70	85
8	IIA	7	80	30	110	70	85
9	IIIC	10	100	10	110	50	70
The mean gain in flexion/extension arc 80 degrees							

a Patient satisfaction after operation on a scale of 1 to 10 with 1 not at all satisfied and 10 very satisfied.

b Mayo elbow performance index.

c Position of fixed flexion deformity in degrees.

d End point of flexion on flexion extension arc.

e Pronation in degrees of the pronation/supination arc.

f Supination in degrees of the pronation supination arc.

## Discussion

Terms heterotopic ossification and myositis ossificans have been used inconsistently and interchangeably for cell-mediated lamellar ectopic bone formation in muscle and soft tissues. Formation of mature lamellar bone confined to muscle is called myositis ossificans, and extra osseous soft tissues is called heterotopic ossification^5^. Hastings *et al *classified HO by the severity of restriction of elbow movements^3^. Class I includes patients with positive radiographs for heterotopic ossification, but no functional limitations. Class II radiographs demonstrate heterotopic ossification, and there is a functional limitation either in the flexion/extension axis (Class IIA), or the pronation/supination axis (Class IIB), or both the axes (Class IIC). Class III patients have the ectopic bone with ankylosis either in flexion/extension (Class IIIA), pronation/supination (Class IIIB), or both (Class IIIC). We had four elbows of class IIIA, one IIIC, four IIA and one IIC in our study.

Ring *et al* reported results of operative excision of complete bony ankylosis in 20 elbows with HO^8^. The etiology of HO was burns in 11 and trauma in nine elbows. They reported comparable results of HO excision in burns and trauma. We did not have any burn patient in our cohort. All except one patient had HO after elbow trauma in our cohort. One patient sustained snake bite and had to be shifted to life support on a ventilator for 56 days. He developed HO of both elbows. The outcomes of the patient above were not different from others^6-8^. The mean MEPI score of our patients was 81. The MEPI score is influenced by elbow stability, pain and activities of daily living besides range of motion of the elbow joint. Recovery of functional range of motion alone is not sufficient for excellent MEPI. Our results for recovery of elbow range of motion are comparable to other studies^6-8^. We assessed pain in ASES-E scores in our cohort of patients. Three of the ten elbows were painless. Seven elbows were having pain either on lifting heavy objects (>5KG) or doing repeated elbow movements. ASES-E scores measure pain while doing different activities. Therefore, patients in our study had pain either on lifting heavy objects or doing repeated elbow movements. This finding has not been reported in other studies. In spite of pain, all patients were able to manage their activities of daily living except one who had a recurrence of HO.

We had 20% (2 of 10 elbows) postoperative complications in our study. We had poor results on one of the elbows with class IIIA HO. The patient did not gain any movement in the elbow joint after the operation because of the recurrence. Another patient with HO class IIIC sustained radial nerve palsy, deep infection and instability of his elbow joint after the operation. His radial nerve palsy recovered but deep infection required repeated surgical procedures. Salazar *et al *in a retrospective study of 46 elbows which underwent surgical excision of HO reported adverse outcomes in preoperative to the final arc of motion of the elbow joint in patients with hypertension, obesity, and absence of per-operative anterior transposition of the ulnar nerve^9^. They reported an average of 17 %( 8 of 46 cases) postoperative complications associated with HO excision. Their complications included three nerve palsies, one deep infection, three HO recurrences and one instability.

We operated patients in our study at the time of presentation. We did not depend on the radiological evidence of maturation of HO in our study. Four patients were operated in six months; two patients were operated in one year, and three patients were operated in two years after the onset of HO. Conventional surgical delay of one year or more for maturation of HO has no basis. This has been demonstrated in various studies from time to time^10, 11^. Chalidis *et al* do not support the concept that early excision triggers the later recurrence^10^. Chen *et al* in a retrospective study of 164 patients over four years studied the time point in surgical excision of heterotopic ossification of the stiff post-traumatic elbow^11^. They recommended early excision with early exercise useful for the treatment of HO aiming at a low recurrence rate and satisfactory function.

We routinely used Indomethacin (dose adjusted as per weight) for duration of six weeks after the operation. Though our sample size was small, we had the recurrence of HO in one of our patients. Various studies have demonstrated the efficacy of NSAID’s for HO prophylaxis after major hip and elbow surgery^12, 13^. Sun *et al* in a retrospective study of 154 patients assessed the efficacy of celecoxib in preventing heterotopic ossification recurrence after open arthrolysis for post-traumatic elbow stiffness in adults^13^. They found a short course of celecoxib to be helpful in the prevention of HO recurrence after open arthrolysis for elbow stiffness in adults and could be an effective and safe option.

The role of radiotherapy for the prophylaxis of HO is controversial. Milakovic *et al* reviewed the 12 randomized controlled trials in a systematic review on the efficacy of radiotherapy for the prophylaxis of heterotopic ossification^14^. They did not find a difference between postoperative or preoperative radiotherapy in preventing HO progression. Ploumis *et al* in a systematic review of studies on radiotherapy for prevention of heterotopic ossification of the elbow reported weak evidence in support of its use^15^. However, we did not use single or multiple doses of radiotherapy in our patients in post-operative period for prophylaxis against recurrent HO.

The choice of surgical approach in our study was based upon the anatomical location of HO. Various studies have recommended the choice of surgical approach, and the technique for excision of HO^8, 9, 16, 17^. We routinely transposed ulnar nerve in all of our cases. Some loss of range of motion is associated with significant injuries of the elbow joint, but the full range of motion is unnecessary for activities of daily living. Functional range of motion of the elbow joint is considered to be from 30 degrees to 130 degrees of flexion, an arc of 100 degrees. Flexion contractures exceeding 45 degrees are unacceptable and severely restrict patient’s ability to perform activities of daily living. We had residual flexion contractures in all elbows after the operation, but most of our patients could perform activities of daily living.

The limitations of our study are its small sample size, retrospective nature and failure to record preoperative subjective and objective scores. To conclude, surgical excision of HO of elbow results in excellent to good functional outcomes.
